# Immunohistochemical Characterization of Procaspase-3 Overexpression as a Druggable Target With PAC-1, a Procaspase-3 Activator, in Canine and Human Brain Cancers

**DOI:** 10.3389/fonc.2019.00096

**Published:** 2019-02-25

**Authors:** Lisa J. Schlein, Bahaa Fadl-Alla, Holly C. Pondenis, Stéphane Lezmi, Charles G. Eberhart, Amy K. LeBlanc, Peter J. Dickinson, Paul J. Hergenrother, Timothy M. Fan

**Affiliations:** ^1^Department of Pathobiology, University of Illinois at Urbana-Champaign, Urbana, IL, United States; ^2^Department of Veterinary Clinical Medicine and Carl R. Woese Institute for Genomic Biology, University of Illinois at Urbana-Champaign, Urbana, IL, United States; ^3^Department of Neuropathology and Ophthalmic Pathology, Johns Hopkins University, Baltimore, MD, United States; ^4^Comparative Oncology Program, Center for Cancer Research, National Cancer Institute, Bethesda, MD, United States; ^5^Department of Surgical and Radiological Sciences, University of California, Davis, Davis, CA, United States; ^6^Department of Chemistry and Carl R. Woese Institute for Genomic Biology, University of Illinois at Urbana-Champaign, Urbana, IL, United States

**Keywords:** glioma, meningioma, brain cancer, procaspase-3, PAC-1, canine comparative

## Abstract

Gliomas and meningiomas are the most common brain neoplasms affecting both humans and canines, and identifying druggable targets conserved across multiple brain cancer histologies and comparative species could broadly improve treatment outcomes. While satisfactory cure rates for low grade, non-invasive brain cancers are achievable with conventional therapies including surgery and radiation, the management of non-resectable or recurrent brain tumors remains problematic and necessitates the discovery of novel therapies that could be accelerated through a comparative approach, such as the inclusion of pet dogs with naturally-occurring brain cancers. Evidence supports procaspase-3 as a druggable brain cancer target with PAC-1, a pro-apoptotic, small molecule activator of procaspase-3 that crosses the blood-brain barrier. Procaspase-3 is frequently overexpressed in malignantly transformed tissues and provides a preferential target for inducing cancer cell apoptosis. While preliminary evidence supports procaspase-3 as a viable target in preclinical models, with PAC-1 demonstrating activity in rodent models and dogs with spontaneous brain tumors, the broader applicability of procaspase-3 as a target in human brain cancers, as well as the comparability of procaspase-3 expressions between differing species, requires further investigation. As such, a large-scale validation of procaspase-3 as a druggable target was undertaken across 651 human and canine brain tumors. Relative to normal brain tissues, procaspase-3 was overexpressed in histologically diverse cancerous brain tissues, supporting procaspase-3 as a broad and conserved therapeutic target. Additionally, procaspase-3 expressing glioma and meningioma cell lines were sensitive to the apoptotic effects of PAC-1 at biologically relevant exposures achievable in cancer patients. Importantly, the clinical relevance of procaspase-3 as a potential prognostic variable was demonstrated in human astrocytomas of variable histologic grades and associated clinical outcomes, whereby tumoral procaspase-3 expression was negatively correlated with survival; findings which suggest that PAC-1 might provide the greatest benefit for patients with the most guarded prognoses.

## Introduction

In 2018, approximately 23,800 adults and 3,560 children in the US were diagnosed with malignant primary brain or spinal cord tumors, and soberingly, 16,830 adult deaths were attributed to inadequate treatment of these primary CNS tumors (1). Approximately 75% of aggressive brain cancers in humans are classified as malignant gliomas and the prognosis for patients with either anaplastic astrocytoma (grade III) or glioblastoma multiforme (GBM; grade IV) is poor due to the invasive nature of these neoplasms. Even with multimodality therapies including surgery and radiochemotherapy, median survival times for patients diagnosed with anaplastic astrocytoma or GBM are less than 36 or 15 months, respectively (2).

Paralleling the aggressive disease course of invasive malignant gliomas, higher grade meningiomas (WHO grades II and III) referred to as atypical and anaplastic, respectively, remain clinically problematic in a subset of human patients. For atypical and anaplastic meningiomas, the likelihood of local recurrence is 29–52% and 50–94%, respectively (3), and is driven by the brain invasive characteristics of these higher grade meningiomas (4–7). Of exceptional gravity are the outcomes for patients diagnosed with anaplastic meningiomas, where the average 5-year survival rates range from 30 to 60% (8, 9).

Since there have been few improvements in the treatment of malignant glial tumors and invasive meningiomas over the past decade, the discovery of new treatments for malignant CNS tumors are needed to improve long term outcomes in these affected patient populations. To identify and validate druggable targets and novel treatment strategies, the inclusion of model systems that most faithfully recapitulate the natural course of brain cancer initiation, promotion, and progression should be included into the therapeutic development path. Collectively, the scientific community has utilized diverse experimental systems and comparative models to advance the study of CNS malignancies, including dogs with spontaneously-arising brain cancer (10–13).

Similar to humans, primary brain tumors are a significant cause of morbidity and mortality in dogs, affecting up to 4.5% of the aged population (14). The most common CNS brain tumors in dogs are meningiomas (~45–50%), gliomas (~40–70%), and choroid plexus neoplasms (~5–7%) (15–18). Treatment options for dogs with brain cancer include surgery, radiation therapy, chemotherapy, or a combination of modalities (19–23). Since the tumor incidence, tumor histologies, and molecular genetic features of canine brain tumors are remarkably similar to their human counterparts, this positions dogs uniquely as translational models for human brain cancer biology and investigational therapeutic research (15, 16, 24–27).

In both humans and companion animals, there is a need to identify effective treatment modalities for brain tumors, especially those that are difficult to resect, with the aims to significantly improve quality of life and survival times. In the age of personalized medicine, ideal therapeutics should target molecular aberrations within the cancer cell population, while sparing normal tissues from potentially harmful side effects. In both the human and veterinary literature, evasion of apoptosis is a common cellular transformation in intracranial neoplasms (28–33). As such, treatment strategies that selectively activate programmed cell death in CNS tumor cells have potential to improve long term outcomes in patients diagnosed with malignant brain cancers.

PAC-1 is a blood-brain barrier (BBB) penetrant, small molecule, pro-apoptotic activator of procaspase-3 (PC-3), that possesses favorable pharmacokinetics, tolerability, and synergistic activities when combined with conventional treatment modalities in animal models of glioma, including naturally-occurring brain cancer in pet dogs (34). Mechanistically, PAC-1 activates PC-3 *in vitro* and in cancer cells through the chelation of inhibitory zinc (35, 36), and based upon PAC-1's binding affinity for zinc (*K*_d_ ~40 nM), selective chelation of labile zinc from PC-3 is achieved in the absence of disrupting the function of proteins containing essential zinc ions (37). Importantly, cellular sensitivity to apoptosis induction with PAC-1 is associated with the resting cellular PC-3 concentration, and given many malignantly transformed cells have elevated PC-3 levels, PAC-1 therapy allows selective induction of apoptosis of cancerous cells (38). While PAC-1 has demonstrated promising preclinical activity in murine and canine models of glioma (34), whether PC-3 is robustly overexpressed and a conserved therapeutic target in naturally-occurring human and canine brain cancer malignancies has not been systematically evaluated. To address this gap in current knowledge, we sought to investigate PAC-1's broader applicability for the treatment of various brain cancer malignancies, as well as to justify the inclusion of pet dogs as a comparative tumor model for PC-3 activating strategies, we performed large-scale validation of PC-3 as a druggable target across 651 human and canine brain tumor samples, evaluated the prognostic significance of PC-3 expressions in human glial tumors, and tested sensitivity of immortalized glioma cell lines to PAC-1 under biologically achievable conditions.

## Materials and Methods

### Cell Lines

Two human glioma cell lines, U118-MG and U87-MG from ATCC (Manassas, VA), and IOMM and KT21 human meningioma cell lines were provided by Gregory Riggins (Johns Hopkins University). Three canine glioma cell lines, SDT-3g, GO6A, and J3T-Bg, were provided by Peter Dickinson (UC Davis). Cells were cultured at 37°C in Dulbecco's Modified Eagle's Medium (DMEM) supplemented with penicillin (100 IU/mL), streptomycin (100 IU/mL), and 10% fetal bovine serum with 5% CO_2_. Cell cultures were maintained in subconfluent monolayers and passaged 2–3 times weekly as needed. Cell lines were tested by STR (short tandem repeat) analysis at the University of Arizona (human) and at the Flint Animal Cancer Center Cell Line Validation Core at Colorado State University (canine).

### Antibodies

Antibodies and their manufacturers, isotypes, pretreatments, dilutions, and times of application are provided in Table 1. Antibodies were purchased from the listed manufacturer or were provided by Stéphane Lezmi.

**Table 1:** Antibodies.

**Antibody**	**Manufacturer**	**Host & Isotype**	**Pre-treatment**	**Dilution**	**Time**	**Staining**
Procaspase-3, #ab32150	Abcam, Cambridge, MA	Rabbit monoclonal IgG	Diva Decloaker	1:3,000	30 min	Automated
			5 min			
Caspase-3, #ab13585	Abcam, Cambridge, MA	Mouse monoclonal IgG1	Diva Decloaker	1:100	60 min	Automated
			5 min			
Calbindin-D, #c9848	Sigma-Aldrich, St. Louis, MO	Mouse monoclonal IgG1	Diva Decloaker	1:3,000	30 min	Automated
			5 min			
Caspase-3, #ab2171	Abcam, Cambridge, MA	Mouse monoclonal IgG2a	Diva Decloaker	1:50	60 min	Automated
			5 min			
GAD67, #mab5406	Millipore, Billerica, MA	Mouse monoclonal IgG2a	Diva Decloaker	1:800	30 min	Automated
			5 min			
Doublecortin, #ab18723	Abcam, Cambridge, MA	Rabbit polyclonal	Citrate 40 min at 87°C	1:3,000	8 hr	Manual
NeuN, #ab177487	Abcam, Cambridge, MA	Rabbit monoclonal	Citrate 30 min at 97°C	1:1,000	30 min	Manual
GFAP, #Z0334	Dako, Santa Clara, CA	Rabbit monoclonal	Trypsin 10 min at 37°C	1:500	30 min	Manual
Green fluorescent antibody, A865	ThermoFisher Scientific, Waltham, MA	Goat anti-Mouse IgG APC	-	1:100	60 min	Manual
Red fluorescent antibody, A11034	ThermoFisher Scientific, Waltham, MA	Goat anti-Rabbit IgG, Alexa Fluor 488	-	1:200	60 min	Manual
Beta Actin, #ab6276-100	Abcam, Cambridge, MA	Mouse monoclonal IgG1	-	1:5,000	30 min	Manual

### Cell Protein Collection

Cells were grown in culture until 80–90% confluent. Cells were washed with PBS and trypsinized, then collected via centrifugation and washed again with PBS. Resultant cell pellets were homogenized with 100 μL of M-PER (Pierce, Rockford, IL), then mixed with fresh Pierce protease inhibitor cocktail solution (diluted 1:100 for final working solution). The homogenate was placed on a shaker for 15 min at room temperature (RT). Cellular protein concentrations were determined using a standard assay kit (BCA, Pierce, Rockford, IL).

### Tissue Collection

Tissues were collected from previously euthanized shelter dogs. Brains were collected within 5 h of death from 21 dogs with no prior history of clinically overt systemic illness. Sections of the cerebral cortex, hippocampus, cerebellum, and brain stem (obex) were taken from each dog; samples from each anatomic area were preserved both in 10% buffered formalin and via flash freezing with dry ice. Frozen samples were stored at −80°C until analysis. Small sections of frozen tissue were weighed and then added to an Eppendorf tube with Tissue Protein Extraction Reagent (T-PER, Pierce, Rockford, IL) at recommended concentrations and manually homogenized. The tissue sample was placed on a shaker for 15 min at RT, then centrifuged. Supernatants were collected and used to determine protein concentration using a standard assay kit (BCA, Pierce, Rockford, IL).

### Western Blot Analysis

For evaluation of procaspase-3, PARP, cleaved PARP, and XIAP expressions, 50 μg samples of cells were electrophoresed on 12% polyacrylamide gel, and then transferred electrophoretically to a nitrocellulose membrane. Beta-actin (Abcam #ab6276-100, Cambridge, MA) was used as a loading control. Band intensity was measured with Image Lab computer software (v. 6.0.0, BioRad Laboratories, Hercules, CA).

### Immunohistochemistry

Formalin-preserved samples were fixed for 24 h, then paraffin-embedded. Immunohistochemical (IHC) staining was performed using an indirect immunoperoxidase technique with diaminobenzidine (DAB) as the chromogen for procaspase-3 (PC-3), caspase-3 (C-3), and isotype controls (GAD67 and Calbindin-D-28K). For these antibodies, IHC staining was performed using an autostainer (intelliPATH FLX, Biocare, Concord, CA). Processed slides were deparaffinized in xylene and rehydrated in alcohol. Endogenous peroxidase activity was blocked with Biocare #PX968 Peroxidazed 1 at RT for 5 min, rinsed with TBS wash buffer, and then incubated for 10 min at RT with Biocare #BP974 Background Punisher. Slides were incubated with procaspase-3 antibody (Abcam #ab32150) for 30 min, washed, and then incubated with Rabbit-on-Canine HRP-Polymer (Biocare #RC542) for 30 min. Slides were washed with TBS, then the reaction was developed using DAB substrate for 5 min. Slides were counterstained with Mayer's hematoxylin. Human tonsil and canine lymph node served as species-specific negative and positive controls.

Additionally, canine brain tissue with moderately intense PC-3 staining was further evaluated using double fluorescent staining, in which PC-3 or PC-3 and caspase-3 (C-3) staining was coupled with doublecortin, GFAP, NeuN, or synaptophysin. Slides were deparaffinized in xylene twice for 5 min. Slides were rehydrated with 100% ethanol, twice for 3 min, and once with 95% ethanol for 1 min. The slides were rinsed in distilled water prior to receiving pre-treatment, as outlined in Table 1. Slides were rinsed in PBS-Tween 20 two times for 2 min. Slides were blocked with 1% BSA and incubated with both primary antibodies for 1 h at RT. Slides were rinsed in PBS-Tween 20 twice for 3 min, incubated with secondary fluorescent antibodies in PBS for 30 min, and then rinsed in PBS-Tween 20 twice for 3 min. Slides were counterstained with DAPI for 20 min at RT, and then rinsed in PBS-Tween 20 twice for 2 min. Slides were cover-slipped with anti-fade fluorescent mounting medium. Canine lymph node served as negative and positive control. Slides were imaged with a Zeiss LSM 700 confocal microscope.

### Immortalized Glioma and Meningioma Cell Sensitivity to PAC-1

All cells were allowed to attach to cell culture plates at least 24 h prior to treatment. A DMSO vehicle control was used in all experiments. Cell viability was assessed using a CellTiter-Blue assay (Promega, Fitchburg, WI). For each cell line evaluated, 2000 cells were seeded in 100 μL of complete media in 96-well plates. Cells were treated with 72-h continuous PAC-1 therapy at doses ranging 0.1–100 μM. Following the study period, cell viability was assessed with the CellTiter-Blue reagent and readout from a fluorescent plate reader. In all cases, dose-response curves and IC_50_ determinations were performed with Origin software (v. 10.4.12.59996, OriginLab, Northampton, MA). For the demonstration of PAC-1 mechanistic activity, immortalized glioma and meningioma cell lines were grown in 6 well plates until confluence, and then exposed to PAC-1 at various concentrations (0 [DMSO vehicle], 6.25, 12.5, 25, and 50 μM) for 24 h prior to whole protein lysate collection and analysis by western blot for PARP and cleaved PARP.

### *In vivo* Activity of PAC-1 for Delaying Intracranial Glioma Growth

All animal procedures were approved by the University of Illinois IACUC (Institutional Animal Care and Use Committee; protocol #15030). 8-week-old female intact C57BL/6 mice were obtained from Charles River. Mice were allowed to acclimate to their new environment at least 7 days prior to cell implantation. The day prior to surgery, mice were anesthetized using 2–3% isoflurane in an induction chamber, then were maintained on 1.5–2% continuous flow isoflurane via a nose cone. A ~1 cm square area was shaved caudal to the orbit and just to the right of midline in preparation for surgery. A small amount of Nair hair removal cream was used to remove residual fur. On the day of surgery, media containing non-adherent GL261 neurospheres was collected. Collected GL261 cells were centrifuged at 1,500 rpm at 4°C for 5 min and viability assessed with trypan blue exclusion. GL261 cells were washed twice with Hanks Balanced Salt Solution (HBSS), then suspended in a solution of 50,000 cells/0.5 μL and placed on ice. Mice were induced and anesthetized as previously described and a 5 mm incision was made slightly to the right of midline and just caudal to the orbit. A Stereotaxic unit was used to place the cellular implantation site +0.55 mm anterior and 2.5 mm to the right of the Bregma. The skull was punctured using a 27 g needle mounted on the Stereotaxic holder. A 0.5 μL Hamilton syringe with a 33 g needle was advanced −3.5 mm ventral to the skull surface. GL261 cells were injected over a period of 1 min, and 2 min were given to allow back pressure to dissipate. The syringe was slowly raised over 30 s. The incision site was closed with a small drop of VetBond. Mice were placed in individual clean cages to allow the incision sites to heal.

Mice were imaged with MRI at days 10 and 29 following GL261 tumor implantation. Data was acquired on a vertical bore imaging scanner (Oxford Instruments, Abington, UK) equipped with a Unity/Inova console (Varian, Palo Alto, CA), operating at 14.1 T and dedicated to small animal studies. A recently patented radiofrequency coil and holder, specifically designed for mouse brain MRI/MRS, was employed to make experimental studies more informative and efficient (B.Odintsov “Tunable Radiofrequency Coil,” US Patent #US 8,049,502 B2; November 1, 2011). Tumor volumes were calculated using ImageJ software. Mice received 10 days of PAC-1 (*n* = 4) or sham therapy (*n* = 4) prior to a follow-up MRI on day 29 post-implantation. Sham therapy mice received HPβCD vehicle control every 12 h via oral gavage, experimental mice received PAC-1 (100 mg/kg) in HPβCD vehicle every 12 h via oral gavage. Oral PAC-1 was administered on a 5 day treatment, 2 day off schedule.

### Scoring of Immunoreactivity Data

Three commercial human microarrays (MG801a, GL2082, and GL2083a, US Biomax, Inc., Rockville, MD) and three additional human microarrays and tissues from normal human hippocampus and medulla (courtesy Charles Eberhart, Johns Hopkins University) were evaluated for PC-3 staining intensity. The use of human tissue samples in the research conducted was approved by the Human Subjects Institutional Review Board at the Johns Hopkins University. Additionally, three canine glioma microarrays (courtesy Peter J. Dickinson, UC-Davis) and 32 archived samples from the University of Illinois Veterinary Diagnostic Laboratory were evaluated for PC-3 immunostaining using ab32150 (Abcam, Cambridge, MA). Samples were assigned a numerical designation, and a random number generator was used to select each sample prior to evaluation.

**Figure 1:**
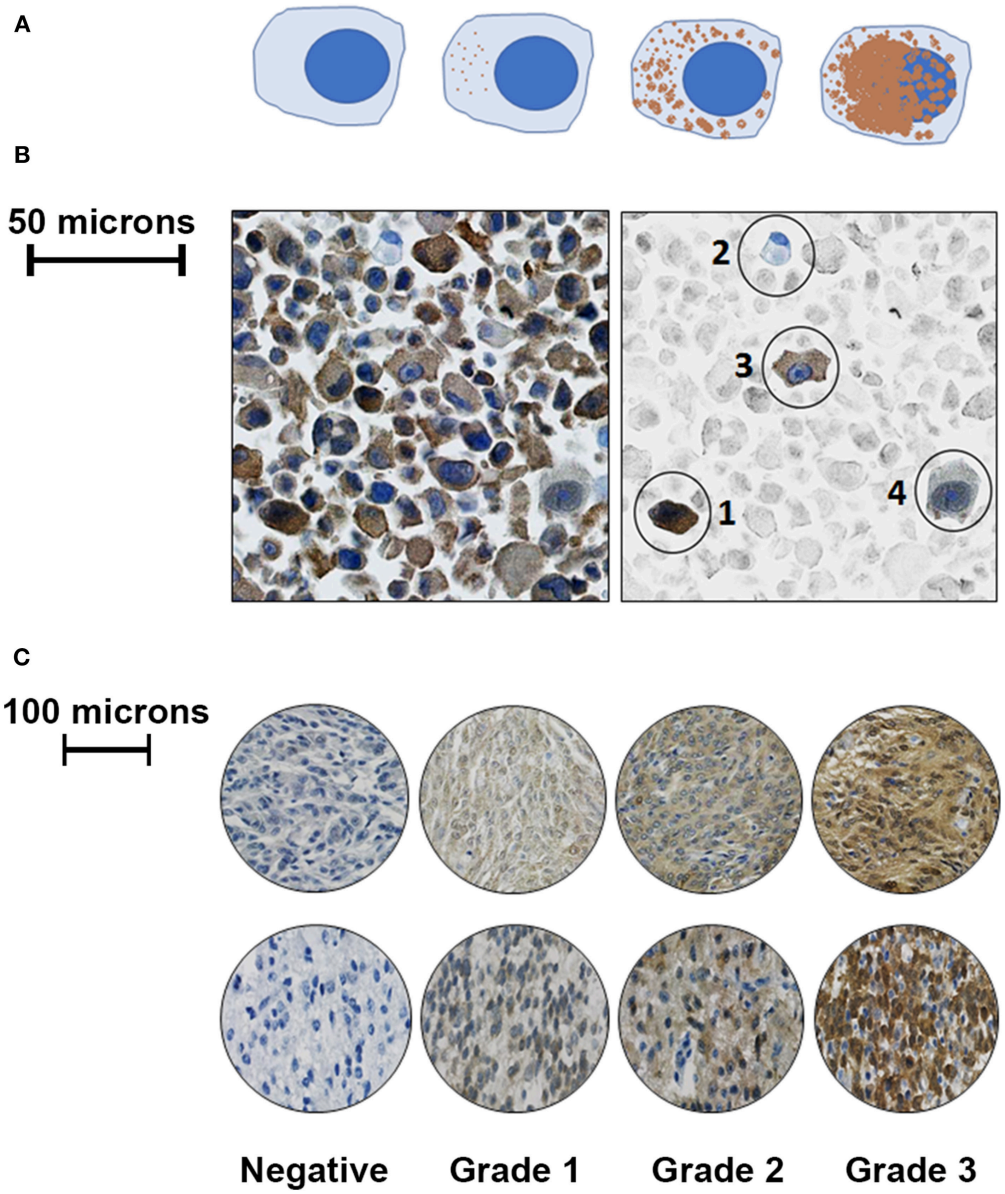
Representative illustrations and images to demonstrate cell grading criteria. **(A)** Left to right- Negative cells contained no cytoplasmic staining for PC-3; faintly positive cells had < 50% cytoplasmic staining; moderately positive cells had ≤ 50% positive cytoplasmic staining; and strongly positive cells had extensive cytoplasmic staining obscuring cellular nuclear detail. **(B)** Left: unaltered view of PC-3 immunostaining from a malignant canine astrocytoma. Right: Same image with representative cells in color, while the background cells are faded; from left to right: (1) a strongly staining cell, (2) a negatively staining cell, (3) a moderately staining cell, and (4) a faintly staining cell. **(C)**: Representative images of canine meningioma samples (top row) and astrocytoma tissue microarray cores (bottom row) that were graded following evaluation of 500 cells using the above criteria.

Five hundred cells—or as many as were available—from each sample were graded by one observer (LJS) on a continuous grading scale, and the percentage of negative, faintly staining, moderately staining, and strongly staining cells were recorded (see Figure 1 for illustration). Negatively staining samples contained < 10% positive cells. Cells that had < 50% cytoplasmic staining were graded as “faintly stained,” those with >50% cytoplasmic staining were graded as “moderately stained,” and those with >50% cytoplasmic staining and in which nuclear detail was obscured by staining intensity were categorized as “strongly stained.” Manual grading was repeated three times for each sample to ensure consistency; although cell percentages differed slightly between observations for the same tumor, the final manual tumor grade was the same in each case.

Microarray samples were secondarily evaluated using the iCyte automated imaging cytometer (Model TLC 1413, ThorLabs, Newton, NJ). The iCyte is a laser-scanning microscope that combines digital imaging with real time population data analysis of analytical cytometry; in additional to detecting fluorescence, the iCyte is able to detect and quantify the amount of DAB chromogen staining. Gates were set using examples of each staining category from manually graded samples. When there were multiple cores of tissue available for a single case, the mean percentages of faintly, moderately, and strongly positive cells were used to determine a final grade for the sample. Normal tissue was used to establish the threshold between negatively and positively staining tumor samples. For the canine samples, sections of normal brain were used as negative controls. For human tissues, microarray cores and tissues (courtesy Charles Eberhart, Johns Hopkins University) were used as negative controls. Raw grading scores were determined using the following formula:

$$\begin{array}{l} 1\;x\;\left(\%\;faintly\;positive\;cells\right)+2\;x\;\left(\%\;moderately\;positive\;cells\right)\\ \;+3\;x\;\left(\%\;strongly\;positive\;cells\right) \end{array}$$

PC-3 immunostaining grades were assigned based on natural numeric cutoffs observed following use of this formula, and tumors were categorized as negative for scores < 10, grade 1 or faintly staining for scores < 50, grade 2 or moderately staining for scores < 150, or grade 3 or strongly staining for scores >150.

## Results

### Procaspase-3 Is Expressed Predictably and at Low Levels in the Normal Human and Canine Brain

**Figure 2:**
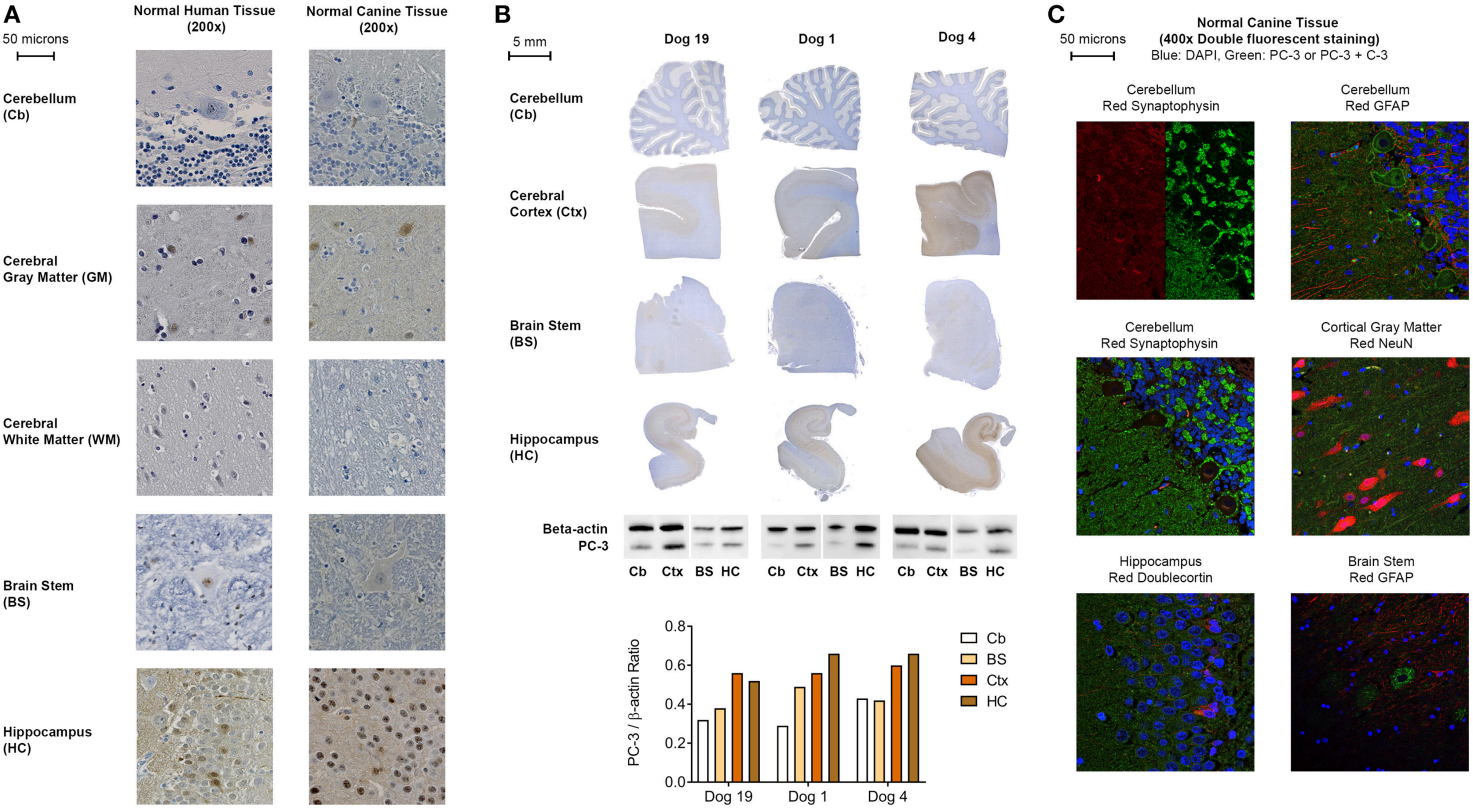
**(A)** Representative PC-3 IHC images from human and canine brain samples (DAB and hematoxylin, 200x). **(B)** Representative PC-3 expressions in normal control dog brains by immunohistochemistry and western blot analysis. Cb, cerebellum; Ctx, cerebral cortex; BS, caudal brain stem (obex); HC, hippocampus. Note increased WB and IHC PC-3 expression in the cortex and hippocampus as compared to the brain stem and cerebellum in all dogs. A consistent IHC staining pattern was seen in all 21 control dogs. **(C)** Representative double fluorescent immunostaining of normal canine brain, 400x. Blue stain: DAPI; Green stain: PC-3 and Caspase-3 (C-3) for all tissues except cerebellum with synaptophysin (PC-3 only); Red stain: clockwise from top left: cerebellum: synaptophysin, cerebellum: GFAP, cortical gray matter: NeuN, brain stem: GFAP, hippocampus: doublecortin, cerebellum: synaptophysin. All canine tissues are from Normal Dog 4, the control dog that exhibited the most intense PC-3 staining.

Based upon our standardized grading scheme (Figure 1), within human control tissues, there was minimal PC-3 staining in human cerebral white matter and cerebellum samples. Staining was generally mild and cytoplasmic, with occasional fine, punctate nuclear staining observed in some areas. There was more intense immunostaining in the cerebral gray matter and hippocampus than in the cerebral white matter or cerebellum (Figure 2A).

Similarly, a comparable PC-3 immunostaining pattern was observed in all normal canine brains (Figure 2A). Macroscopically, in the five anatomic regions sampled, PC-3 expression appeared strongest in the hippocampus, particularly in the dentate gyrus, and in the cerebral cortical gray matter (Figure 2B). Likewise, there was less intense immunostaining in the brain stem and cerebellum. Immunoblotting for PC-3 using flash-frozen sections of canine brain corroborated these IHC findings (Figure 2B). Although the staining pattern in these various anatomic areas was consistent among the dogs sampled, there was subtle inter-individual variability in the PC-3 immunostaining intensity. By confocal fluorescent microscopy, subcellular localization of PC-3 staining was identified to be occasionally within neurons, and consistently in synaptic-like structures on the neuronal membranes, dendrites and axons throughout the normal canine brain (Figure 2C).

### Procaspase-3 Is Overexpressed in Intracranial Neoplasms

**Figure 3:**
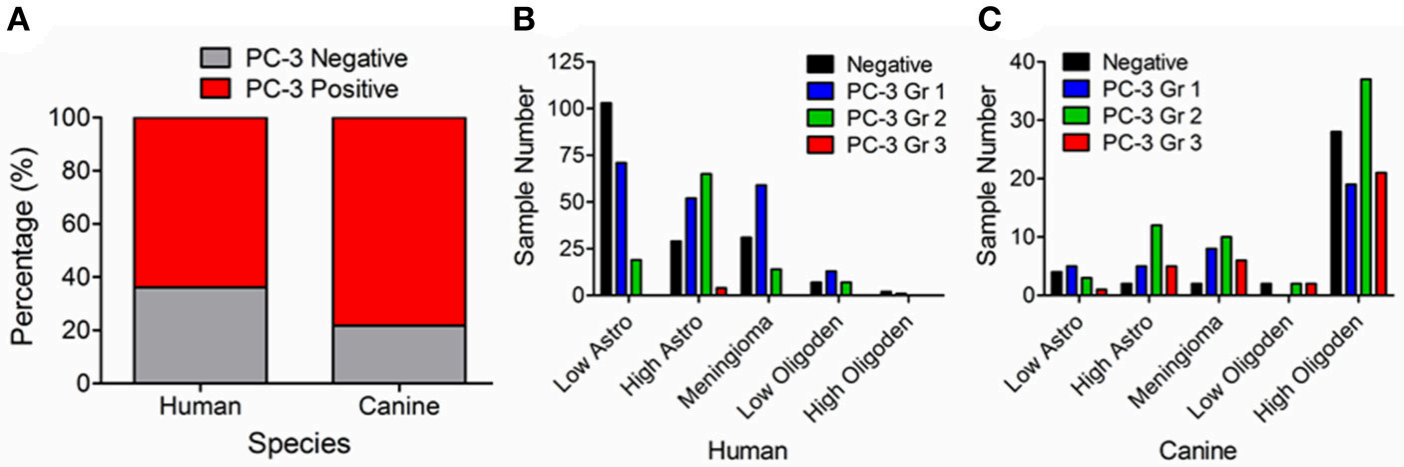
Graphical summary, PC-3 immunoreactivity for all tumor histologies evaluated. **(A)** Most intracranial neoplasms in both humans and dogs overexpress PC-3 (red shading). Identified PC-3 grading patterns in **(B)** 477 primary brain tumors in humans and **(C)** 174 primary brain tumors in dogs. Note that high-grade astrocytomas tend to stain more intensely for PC-3 than their less malignant counterparts in both species.

In 477 human and 174 canine tumors, PC-3 was overexpressed based on IHC relative to normal brain tissue (Figure 3A). Specifically, 62% (211/343) of human astrocytomas and 83% (31/37) of canine astrocytomas overexpressed PC-3. Within this subset of tumors, low histologic grade astrocytomas had less intense PC-3 expression as compared to high histologic grade astrocytomas in both species (Figures 3B,C). 70% (73/104) of human meningiomas and 92% (24/26) of canine meningiomas overexpressed PC-3, while 70% (21/30) of human oligodendrogliomas and 73% (81/111) of canine oligodendrogliomas overexpressed PC-3 (Figures 3B,C, respectively). In 32 cases of primary canine brain tumors from archived cases at the University of Illinois Veterinary Diagnostic Laboratory, 87.5% (28/32) of tumors overexpressed PC-3 relative to the normal brain tissue obtained from cadaver dogs.

A Kappa statistic, which measures inter-observer variation, was >0.81 for both human and canine samples, consistent with almost perfect agreement between manual and iCyte automated cytometer tumor grading (39). For human samples, Kappa = 0.92 (95% confidence interval: 0.89–0.95), and for canine samples, Kappa = 0.88 (95% confidence interval: 0.84–0.92). For all samples considered, Kappa = 0.90 (95% confidence interval: 0.88–0.93).

### Immortalized Glioma and Meningioma Cell Lines Are Sensitive to PAC-1 Therapy at Biologically-Relevant Concentrations

**Figure 4:**
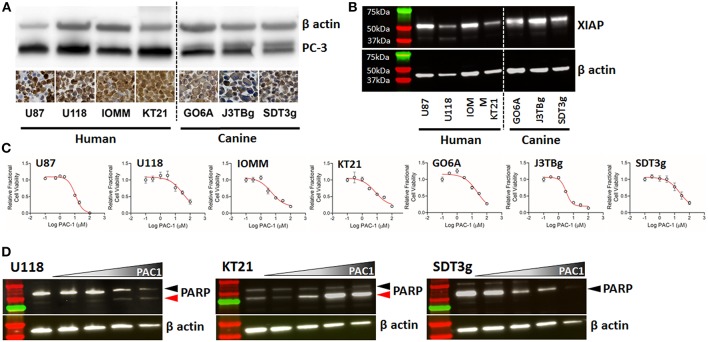
**(A)** Western blots and immunohistochemistry of two human glioma cell lines (U87, U118), two human meningioma cell lines (IOMM and KT21), and three canine glioma cell lines (GO6A, J3TBg, SDT3g), showing positive expression of PC-3; cell pellet immunostaining with Abcam ab32150; 200x, DAB and hematoxylin. Cell pellet images correlate with the cell lines shown in the Western blot. **(B)** Expression of XIAP detected by western blot across human and canine cell lines. **(C)** Representative dose-response curves for each cell line demonstrating conserved sensitivity to PAC-1 in culture at biologically-relevant concentrations and durations of exposure. **(D)** Processing of caspase-3 downstream substrate, PARP, in 3 representative cell lines (human- U118 and KT21; canine SDT3g) following exposure to PAC-1 for 24 h at 0, 6.25, 12.5, 25, and 50 μM (gradient). Variable processing of PARP represented by PARP degradation only (SDT3g), generation of cleaved PARP only (KT21), or combination (U118). Black arrowhead (PARP); red arrowhead (cleaved PARP).

Western blot evaluation across human and canine brain cancer cell lines showed expression of a 32 kDa protein product consistent with PC-3 (Figure 4A). Corroborating the western blot findings, IHC-stained cell pellets derived from the same cell lines also identified robust positive staining for PC-3 in both human and dog-derived cell lines. In addition to PC-3, the expression of XIAP, a member of the inhibitor of apoptosis family proteins (IAP) that negatively regulate executioner caspases, was identified in all cell lines (Figure 4B). *In vitro*, PAC-1 induced cell death in all immortalized cell lines at biologically-relevant concentrations; Figure 4C shows representative IC_50_ dose response curves for individual cell lines, and Table 2 shows aggregate IC_50_ data from 72 h of continuous exposure to PAC-1 for all human and canine cell lines. There was no correlation identified between IC_50_ values and basal expressions of PC-3, XIAP, or the ratio of PC-3/XIAP across the cell lines (data not shown). Supporting PAC-1's mechanism of action, exposures to PAC-1 for 24 h across a range of concentrations (vehicle, 6.25, 12.5, 25, and 50 μM) demonstrates consistent activation of caspase-3 activities represented by either degradation of PARP, accumulation of cleaved PARP, or the combination (Figure 4D).

**Table 2:** Cell lines used in this study.

**Cell Line**	**Species**	**Cell line type**	**72h PAC-1 IC50 (μM)**
U87-MG	Human	Glioma	15.2 ± 2.8
U118-MG	Human	Glioma	19.9 ± 7.7
IOMM	Human	Meningioma	4.25 ± 0.7
KT21	Human	Meningioma	6.5 ± 0.5
GO6A	Canine	Glioma	14.9 ± 6.1
J3TBg	Canine	Glioma	3.3 ± 0.1
SDT3g	Canine	Glioma	10.7 ± 3.7

### PAC-1 Attenuates Growth of Murine GL261 glioma

**Figure 5:**
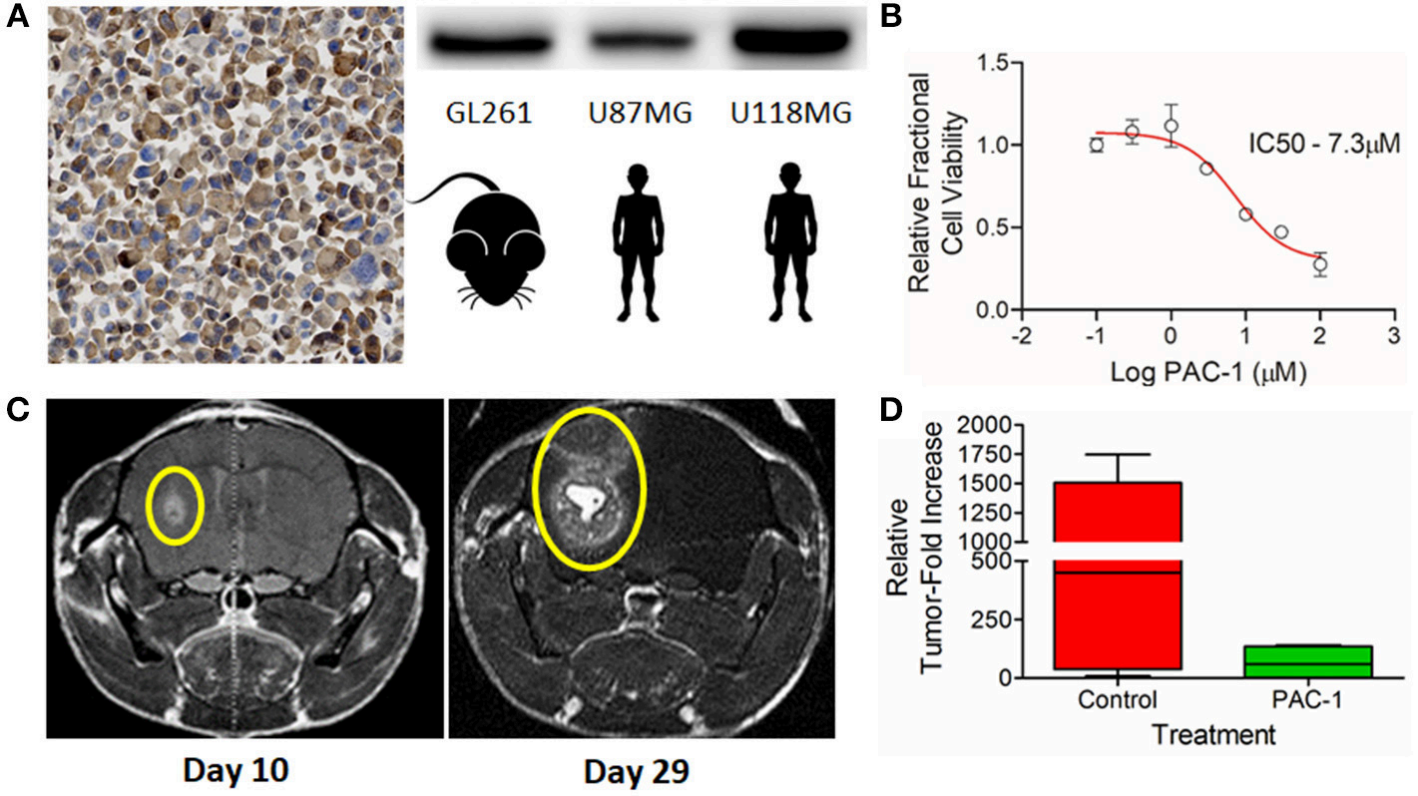
**(A)** Immunohistochemical and western blot detection of PC-3 in the murine glioma cell line, GL261; positive human controls U87MG and U118MG included as comparative references. **(B)** GL261 *in vitro* dose-dependent sensitivity to PAC-1 following 72 h of exposure. **(C)** Reliable induction of orthotopic tumors 10 days following the stereotactic-guided intracranial injection of 50,000 GL261 neurospheres (Left) with subsequent rapid growth over a period of 19 days (Right); gadolinium contrast enhancing tumors identified with yellow ellipses. **(D)** Variable attenuation in GL261 orthotopic growth in mice receiving oral PAC-1 (100 mg/kg) twice daily for 10 treatments compared to sham vehicle treated mice; difference not statistically significant.

The murine glioma cell line, GL261, robustly expresses PC-3 (Figure 5A) and is sensitive to the apoptosis inducing activities of PAC-1 at low micromolar concentrations (Figure 5B). Intracranial implantation of 50,000 GL261 neurospheres into C57BL/6 female mice generates with high penetrance variably sized gadolinium contrast enhancing tumors 10-days following implantation (Figure 5C, left). The median and range of GL261 tumor volumes prior to treatment (day 10) were similar between sham vehicle (1.5 mm^3^; range 0.02–3.1 mm^3^) and PAC-1 (1.5 mm^3^; range 0.09–3.4 mm^3^) treated mice. Over a course of 19 days, GL261 tumors grow rapidly into large, spacing occupying lesions (Figure 5C, right); with the median GL261 tumor volumes in sham vehicle treated mice being 449.2 mm^3^ (range 9.1–1747.6 mm^3^) compared to PAC-1 treated mice being 59.5 mm^3^ (range 1.3–141.2 mm^3^; Figure 5D); however, the relative tumor-fold increases failed to reach statistical significance.

### Procaspase-3 IHC Expression Correlates With Tumor Histologic Grade and Survival

**Figure 6:**
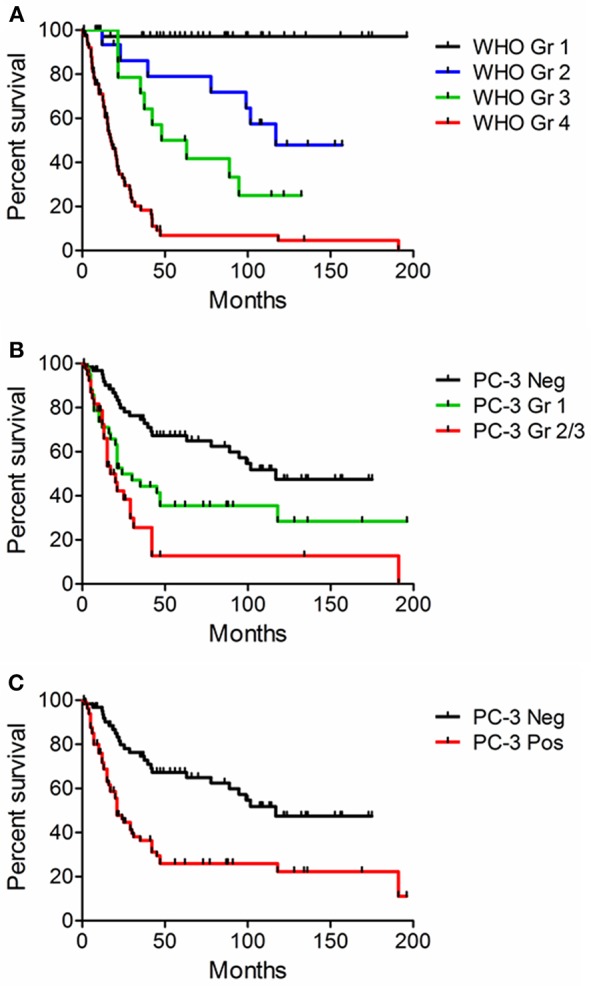
Kaplan-Meier curves. Survival was correlated with WHO histologic grade and PC-3 expression in 157 human glioma samples from tissue microarrays. Log-Rank statistical calculation was performed for all curves and *p* was < 0.001 in each case. **(A)** Survival and WHO histologic Grade. **(B)** Survival and PC-3 immunostaining grade. **(C)** Survival and the presence or absence of positive PC-3 grade. Note that higher grade PC-3 staining is associated with decreased survival.

Across CNS malignant histologies in humans, PC-3 expression was strongest in high-grade astrocytomas. To investigate whether PC-3 IHC grading could correlate with survival in human patients diagnosed with astrocytomas, a subset of 157 samples from Johns Hopkins University with clinical outcome-linked data available were evaluated. Survival correlated, as expected, with WHO histologic grade (Figure 6A). Importantly, PC-3 expressions, either graded or dichotomously categorized (Figure 6B or Figure 6C, respectively), correlated with decreased patient survival (Table 3). Log rank tests were statistically significant in all cases (*p* < 0.001).

**Table 3:** WHO histologic grade, survival, and PC-3 grade.

**WHO histologic grade**	**Grade 1**	**Grade 2**	**Grade 3**	**Grade 4**
# Cases	38	19	14	86
% Censored	97.4%	63.2%	28.6%	26.7%
MST (Months)	N/A	58.7	37.5	15.3
**PC-3 grade**	**Negative**	**Grade 1**	**Grade 2 or 3**	
# Cases	67	45	45	
% Censored	59.7%	42.2%	37.8%	
MST (Months)	24	17	15.3	
**PC-3 staining**	**Negative**	**Positive**	
# Cases	54	66	
% Censored	51.8%	18.2%	
MST (Months)	25.4	15.3	

## Discussion

Malignant gliomas in humans remain clinically challenging to treat given the invasive nature of tumor cells into normal surrounding brain parenchyma, which precludes the feasibility of complete surgical resection in most affected patients. As such, conventional standard-of-care therapy for malignant gliomas in humans is trimodal in nature, inclusive of maximal surgical resection without the creation of unacceptable neurologic deficits, definitive radiation therapy, and adjuvant chemotherapy (2). Despite a modest therapeutic advance following the introduction of temozolomide therapy in 2005, the median survival time in humans with GBM receiving trimodal therapy is disappointingly short, and has remained static at only 14 months (40). To potentially improve outcomes for patients diagnosed with GBM, new therapies which are capable of penetrating into the central nervous system compartment and that preferentially induce apoptosis of cancer cells must be identified.

Resistance to normal cellular apoptosis is a hallmark of tumorigenesis, and there are numerous intracellular proteins—including death receptors, mitochondrial pore proteins, and TP53, that can be upregulated or downregulated, leading to inhibition of normal cellular apoptosis in cancer cells (41). Many efforts have been made to create therapeutics that target dysregulated molecular targets in the apoptotic cascade, yet none have clinically improved outcomes in people with aggressive astrocytomas, such as GBM. Regardless of where upstream cellular mutations occur, both extrinsic and intrinsic arms of the apoptotic cascade converge on the activation of procaspase-3 (PC-3) to caspase-3, the key executioner protease within the cell. Therefore, as a BBB penetrant PC-3 activator, PAC-1 is uniquely positioned as a therapeutic that can circumvent the wide range of upstream apoptotic evasion strategies adopted by cancer cells; and molecularly, it functions via direct chelation of labile cellular zinc with consequent caspase-3 activation and induction of apoptotic cell death (35, 42). While PAC-1 possesses mechanistic advantage for directly activating caspase-3, the existence and dysregulation of IAP family of proteins, such as XIAP residing downstream of executioner caspases that can directly neutralize activated executioner caspases via proteasome degradation (43), could attenuate the selective anticancer apoptotic properties of PAC-1.

While conceptually attractive to induce programmed cell death through direct PC-3 activation, a concern of using a potent pro-apoptotic compound to treat brain cancer is the potential for off-target effects, resulting in cell death in normal brain tissue. Indeed, PC-3 is ubiquitously expressed in the body, and within the developing nervous system, there is an obligate need for programmed cell death to remove unnecessary neurons and for pruning of neuronal synapses (44, 45). Additionally, caspase-3 activity in C57BL/6 mice has been shown to be important in cognition and behavior, particularly inhibitory control, and is a key player in synaptic homeostasis (46). In the current study, PC-3 was identified in normal brain structures in both humans and dogs, albeit at much lower expression levels in comparison to malignantly transformed tissues. By immunofluorescence, PC-3 was identified in synaptic-like structures, and it is plausible that this observed localization may indicate participation in synaptic homeostasis, as identified in mice, in higher mammalian species as well. Despite low expressions of PC-3 identified in normal brain tissues, the observation for histopathologic off-target effects in brain tissues have not been observed in preclinical toxicity studies with healthy rodents receiving orally administered PAC-1 at clinically relevant dosages. More importantly and translationally relevant, PAC-1 has been well-tolerated clinically and neurologically in dogs with spontaneous cancers including gliomas, as well as in humans diagnosed with diverse tumor histologies (34, 47, 48).

In this study, the potential for PAC-1-mediated PC-3 activation is very pertinent to human oncology, as PAC-1 induces cell death in cancer cells in proportion to the resting PC-3 concentration within the cell (38), and our findings support PC-3's overexpression in the majority of both human and canine brain tumors relative to normal tissues. Given the observed differential in PC-3 expressions, it remains plausible that a therapeutic window exists whereby PAC-1 would preferentially induce apoptosis in malignantly transformed cells, yet spare surrounding brain tissues with substantively lower PC-3 expressions. To guide the identification of a therapeutic PAC-1 concentration range for brain cancer therapy, we showed that low micromolar concentrations of PAC-1 lead to cell death across a panel of immortalized cell lines from both species. However, sensitivity to PAC-1 *in vitro* was not identified to be directly proportional to resting PC-3 alone or in the context of XIAP, a known IAP involved in glioma apoptosis resistance (33, 49, 50). Given the multitude of cellular parameters that might influence apoptosis susceptibility and resistance, these findings are not completely unexpected, but rather underscore the complexity of opposing cellular death promoting- and resisting- pathways. Nonetheless and importantly, these *in vitro* concentrations of PAC-1 that reliably induced cancer cell apoptosis are readily achievable in rodents, dogs, and human beings *in vivo* (48, 51). While our *in vitro* data support the role of PAC-1 as a single agent, the greatest clinical benefit to brain cancer patients will likely be achieved through the inclusion of PAC-1 as an adjuvant therapy, a supposition supported by PAC-1's demonstrated ability to be safely combined and synergize with diverse agents, including radiation therapy, other apoptosis-activating agents, and temozolomide (34, 47, 52).

In addition to PC-3 serving as an attractive therapeutic target, our findings provide preliminary support for the prognostic significance of PC-3 for some brain tumor pathologies. In 157 human astrocytoma samples with outcome linked clinical information, increased expression of PC-3 correlated with increasing histologic grade and decreasing survival time. These observed correlations might have two profound clinical implications. First, because some astrocytomas do not express PC-3 (67 out of 157 samples), there would be molecular justification to stratify cohorts of patients to receive or not receive PAC-1 based upon PC-3 expressions, similar to what is already conventionally practiced for the institution of temozolomide based upon tumoral MGMT hypermethylation status. Second, given the inverse correlation between PC-3 and survival time, these findings imply that PC-3 activating strategies might provide the greatest potential benefit in patients with the gravest prognoses, and adjuvant PAC-1 therapies might maximally extend survival times when instituted early in the planned treatment course for affected patients.

Pet dogs with spontaneous tumors provide a unique comparative opportunity to model human disease, including certain types of brain cancer. Dogs often develop spontaneous brain tumors that are histologically indistinguishable from those seen in humans, and can afford the scientific community with a unique model system to study and characterize novel treatment strategies or devices for improving brain cancer management (10, 16, 25, 34). While significant justification exists for the inclusion of comparative oncology for expediting drug development efforts, there are some recognized limitations in using canines as a model of human brain cancer. For example, in humans but not in dogs, meningioma COX-2 expression correlates with proliferative index and tumor grade (53, 54), exonic p53 mutations are more common in human than in canine astrocytomas (55), and there is no correlation between meningioma grade and NF2 expression in canine tumors as is seen in humans (56). As such, leveraging the unique aspects of comparative oncology should be tailored to the most appropriate disease pathologies, and not considered a “catch all” parallel modeling system.

Given the aggregate data generated in our study, PC-3 appears to be a valid and druggable target for specific brain tumor histologies, particularly high grade astrocytomas; and small molecule activators of PC-3, such as PAC-1, should be further explored for their clinical utility for improving the management of brain tumors overexpressing PC-3. Several chemical and pharmacologic properties of PAC-1 are attractive for the management of brain cancer including its oral administrative route, achievement of predicted therapeutic concentrations which are safe in humans and dogs (34, 47, 48, 51), and ability to traverse the BBB (36). Collectively, these data and properties of PAC-1 have led to the conductance of a Phase 1b trial of PAC-1 plus temozolomide in refractory glioblastoma and anaplastic astrocytoma patients (NCT02355525). Although additional study and *in vivo* modeling are needed, these initial data show great promise for PAC-1 as a therapeutic for intracranial neoplasms and for the inclusion of pet dogs with brain cancer as a unique modeling resource for the scientific community.

## Ethics Statement

This study utilized archived human and canine patient samples for histologic assessment, no living patient (human or canine) were used for this study. All human samples were deidentified.

## Author Contributions

PH and TF: project conception and supervision of work. TF, LS, and SL: experimental design. LS, HP, CE, and PD: sample preparation. LS, BF-A, HP, and SL: experimental procedures. LS and TF: prepared the manuscript with support from AL.

### Conflict of Interest Statement

PH and TF have a financial interest in the compound PAC-1 through intellectual property, and both serve on the scientific advisory board of Vanquish Oncology LLC. These potential conflicts are managed through the University of Illinois conflict of interest policy. The remaining authors declare that the research was conducted in the absence of any commercial or financial relationships that could be construed as a potential conflict of interest.

## References

[1] American Society of Clinical Oncology Brain Tumor: Statistics 2018 (2018). Available online at: www.cancer.net/cancer-types/brain-tumor/statistics.

[2] Wen PY Kesari S. Malignant gliomas in adults. N Engl J Med. (2008) 359:492–507. 10.1056/NEJMra0708126 18669428

[3] Champeaux C Dunn L. World Health Organization Grade II meningioma: a 10-year retrospective study for recurrence and prognostic factor assessment. World Neurosurg. (2016) 89:180–6. 10.1016/j.wneu.2016.01.055 26850975

[4] Spille DC Hess K Sauerland C Sanai N Stummer W Paulus W. Brain invasion in meningiomas: incidence and correlations with clinical variables and prognosis. World Neurosurg. (2016) 93:346–54. 10.1016/j.wneu.2016.06.055 27344043

[5] Vranic A Popovic M Cor A Prestor B Pizem J. Mitotic count, brain invasion, and location are independent predictors of recurrence-free survival in primary atypical and malignant meningiomas: a study of 86 patients. Neurosurgery (2010) 67:1124–32. 10.1227/NEU.0b013e3181eb95b7 20881577

[6] Yang SY Park CK Park SH Kim DG Chung YS Jung HW. Atypical and anaplastic meningiomas: prognostic implications of clinicopathological features. J Neurol Neurosurg Psychiatry (2008) 79:574–80. 10.1136/jnnp.2007.121582 17766430

[7] Yoon H Mehta MP Perumal K Helenowski IB Chappell RJ Akture E. Atypical meningioma: randomized trials are required to resolve contradictory retrospective results regarding the role of adjuvant radiotherapy. J Cancer Res Ther. (2015) 11:59–66. 10.4103/0973-1482.148708 25879338

[8] Kaley T Barani I Chamberlain M McDermott M Panageas K Raizer J. Historical benchmarks for medical therapy trials in surgery- and radiation-refractory meningioma: a RANO review. Neuro Oncol. (2014) 16:829–40. 10.1093/neuonc/not330 24500419 PMC4022224

[9] Preusser M Berghoff AS Hottinger AF. High-grade meningiomas: new avenues for drug treatment? Curr Opin Neurol. (2013) 26:708–15. 10.1097/WCO.0000000000000035 24184974

[10] LeBlanc AK Mazcko C Brown DE Koehler JW Miller AD Miller CR. Creation of an NCI comparative brain tumor consortium: informing the translation of new knowledge from canine to human brain tumor patients. Neuro Oncol. (2016) 18:1209–18. 10.1093/neuonc/now051 27179361 PMC4999002

[11] Miyai M Tomita H Soeda A Yano H Iwama T Hara A. Current trends in mouse models of glioblastoma. J Neurooncol. (2017) 135:423–32. 10.1007/s11060-017-2626-2 29052807 PMC5700231

[12] Rao SS Lannutti JJ Viapiano MS Sarkar A Winter JO. Toward 3D biomimetic models to understand the behavior of glioblastoma multiforme cells. Tissue Eng Part B Rev. (2014) 20:314–27. 10.1089/ten.teb.2013.0227 24044776 PMC4128251

[13] Wang C Tong X Yang F. Bioengineered 3D brain tumor model to elucidate the effects of matrix stiffness on glioblastoma cell behavior using PEG-based hydrogels. Mol Pharm. (2014) 11:2115–25. 10.1021/mp5000828 24712441

[14] Song RB Vite CH Bradley CW Cross JR. Postmortem evaluation of 435 cases of intracranial neoplasia in dogs and relationship of neoplasm with breed, age, and body weight. J Vet Int Med. (2013) 27:1143–52. 10.1111/jvim.12136 23865437

[15] Dickinson PJ. Advances in diagnostic and treatment modalities for intracranial tumors. J Vet Intern Med. (2014) 28:1165–85. 10.1111/jvim.12370 24814688 PMC4857954

[16] Hicks J Platt S Kent M Haley A. Canine brain tumours: a model for the human disease? Vet Comp Oncol. (2017) 15:252–72. 10.1111/vco.12152 25988678

[17] Rossmeisl JH Jr. Jones JC Zimmerman KL Robertson JL. Survival time following hospital discharge in dogs with palliatively treated primary brain tumors. J Am Vet Med Assoc. (2013) 242:193–8. 10.2460/javma.242.2.193 23276095

[18] Snyder JM Shofer FS Van Winkle TJ Massicotte C. Canine intracranial primary neoplasia: 173 cases (1986-2003). J Vet Intern Med. (2006) 20:669–75. 10.1111/j.1939-1676.2006.tb02913.x 16734106

[19] Dolera M Malfassi L Bianchi C Carrara N Finesso S Marcarini S. Frameless stereotactic radiotherapy alone and combined with temozolomide for presumed canine gliomas. Vet Comp Oncol. (2018) 16:90–101. 10.1111/vco.12316 28643878

[20] Hu H Barker A Harcourt-Brown T Jeffery N. Systematic review of brain tumor treatment in dogs. J Vet Intern Med. (2015) 29:1456–63. 10.1111/jvim.13617 26375164 PMC4895648

[21] Klopp LS Rao S. Endoscopic-assisted intracranial tumor removal in dogs and cats: long-term outcome of 39 cases. J Vet Intern Med. (2009) 23:108–15. 10.1111/j.1939-1676.2008.0234.x 19175729

[22] Mariani CL Schubert TA House RA Wong MA Hopkins AL Barnes Heller HL. Frameless stereotactic radiosurgery for the treatment of primary intracranial tumours in dogs. Vet Comp Oncol. (2015) 13:409–23. 10.1111/vco.12056 24007303

[23] Zwingenberger AL Pollard RE Taylor SL Chen RX Nunley J Kent MS. Perfusion and volume response of canine brain tumors to stereotactic radiosurgery and radiotherapy. J Vet Intern Med. (2016) 30:827–35. 10.1111/jvim.13945 27149650 PMC4867273

[24] Debinski W Dickinson P Rossmeisl JH Robertson J Gibo DM. New agents for targeting of IL-13RA2 expressed in primary human and canine brain tumors. PLoS ONE (2013) 8:e77719. 10.1371/journal.pone.0077719 24147065 PMC3797726

[25] Dickinson PJ LeCouteur RA Higgins RJ Bringas JR Larson RF Yamashita Y. Canine spontaneous glioma: a translational model system for convection-enhanced delivery. Neuro Oncol. (2010) 12:928–40. 10.1093/neuonc/noq046 20488958 PMC2940703

[26] Rossmeisl JH Jr. Garcia PA Pancotto TE Robertson JL Henao-Guerrero N Neal RE II. Safety and feasibility of the NanoKnife system for irreversible electroporation ablative treatment of canine spontaneous intracranial gliomas. J Neurosurg. (2015) 123:1008–25. 10.3171/2014.12.JNS141768 26140483

[27] Rossmeisl JH Hall-Manning K Robertson JL King JN Davalos RV Debinski W. Expression and activity of the urokinase plasminogen activator system in canine primary brain tumors. Onco Targets Ther. (2017) 10:2077–85. 10.2147/OTT.S132964 28442916 PMC5396930

[28] Courtay-Cahen C Platt SR De Risio L Starkey MP. Preliminary analysis of genomic abnormalities in canine meningiomas. Vet Comp Oncol. (2008) 6:182–92. 10.1111/j.1476-5829.2008.00159.x 19178678

[29] Goldar S Khaniani MS Derakhshan SM Baradaran B. Molecular mechanisms of apoptosis and roles in cancer development and treatment. Asian Pac J Cancer Prev. (2015) 16:2129–44. 10.7314/APJCP.2015.16.6.2129 25824729

[30] Guan H Song L Cai J Huang Y Wu J Yuan J. Sphingosine kinase 1 regulates the Akt/FOXO3a/Bim pathway and contributes to apoptosis resistance in glioma cells. PLoS ONE (2011) 6:e19946. 10.1371/journal.pone.0019946 21625639 PMC3097221

[31] Stegh AH Chin L Louis DN DePinho RA. What drives intense apoptosis resistance and propensity for necrosis in glioblastoma? A role for Bcl2L12 as a multifunctional cell death regulator. Cell Cycle (2008) 7:2833–9. 10.4161/cc.7.18.6759 18769159

[32] Wong RS. Apoptosis in cancer: from pathogenesis to treatment. J Exp Clin Cancer Res. (2011) 30:87. 10.1186/1756-9966-30-87 21943236 PMC3197541

[33] Ziegler DS Kung AL Kieran MW. Anti-apoptosis mechanisms in malignant gliomas. J Clin Oncol. (2008) 26:493–500. 10.1200/JCO.2007.13.9717 18202424

[34] Joshi AD Botham RC Schlein LJ Roth HS Mangraviti A Borodovsky A. Synergistic and targeted therapy with a procaspase-3 activator and temozolomide extends survival in glioma rodent models and is feasible for the treatment of canine malignant glioma patients. Oncotarget (2017) 8:80124–38. 10.18632/oncotarget.19085 29113289 PMC5655184

[35] Peterson QP Goode DR West DC Ramsey KN Lee JJ Hergenrother PJ. PAC-1 activates procaspase-3 *in vitro* through relief of zinc-mediated inhibition. J Mol Biol. (2009) 388:144–58. 10.1016/j.jmb.2009.03.003 19281821 PMC2714579

[36] West DC Qin Y Peterson QP Thomas DL Palchaudhuri R Morrison KC. Differential effects of procaspase-3 activating compounds in the induction of cancer cell death. Mol Pharm. (2012) 9:1425–34. 10.1021/mp200673n 22486564 PMC3348238

[37] Sjoli S Solli AI Akselsen O Jiang Y Berg E Hansen TV. PAC-1 and isatin derivatives are weak matrix metalloproteinase inhibitors. Biochim Biophys Acta (2014) 1840:3162–9. 10.1016/j.bbagen.2014.07.011 25046380

[38] Putt KS Chen GW Pearson JM Sandhorst JS Hoagland MS Kwon JT. Small-molecule activation of procaspase-3 to caspase-3 as a personalized anticancer strategy. Nat Chem Biol. (2006) 2:543–50. 10.1038/nchembio814 16936720

[39] Viera AJ Garrett JM. Understanding interobserver agreement: the kappa statistic. Fam Med. (2005) 37:360–3. 15883903

[40] Delgado-Lopez PD Corrales-Garcia EM. Survival in glioblastoma: a review on the impact of treatment modalities. Clin Transl Oncol. (2016) 18:1062–71. 10.1007/s12094-016-1497-x 26960561

[41] Hanahan D Weinberg RA. Hallmarks of cancer: the next generation. Cell (2011) 144:646–74. 10.1016/j.cell.2011.02.013 21376230

[42] Peterson QP Hsu DC Goode DR Novotny CJ Totten RK Hergenrother PJ. Procaspase-3 activation as an anti-cancer strategy: structure-activity relationship of procaspase-activating compound 1 (PAC-1) and its cellular co-localization with caspase-3. J Med Chem. (2009) 52:5721–31. 10.1021/jm900722z 19708658 PMC2749958

[43] Deveraux QL Takahashi R Salvesen GS Reed JC. X-linked IAP is a direct inhibitor of cell-death proteases. Nature (1997) 388:300–4. 10.1038/40901 9230442

[44] D'Amelio M Cavallucci V Cecconi F. Neuronal caspase-3 signaling: not only cell death. Cell Death Differ. (2010) 17:1104–14. 10.1038/cdd.2009.180 19960023

[45] Erturk A Wang Y Sheng M. Local pruning of dendrites and spines by caspase-3-dependent and proteasome-limited mechanisms. J Neurosci. (2014) 34:1672–88. 10.1523/JNEUROSCI.3121-13.2014 24478350 PMC6827581

[46] Lo SC Wang Y Weber M Larson JL Scearce-Levie K Sheng M. Caspase-3 deficiency results in disrupted synaptic homeostasis and impaired attention control. J Neurosci. (2015) 35:2118–32. 10.1523/JNEUROSCI.3280-14.2015 25653368 PMC6705356

[47] Botham RC Roth HS Book AP Roady PJ Fan TM Hergenrother PJ. Small-molecule procaspase-3 activation sensitizes cancer to treatment with diverse chemotherapeutics. ACS Cent Sci. (2016) 2:545–59. 10.1021/acscentsci.6b00165 27610416 PMC4999974

[48] Danciu OC Nicholas MK Holdhoff M Venepalli NK Hergenrother PJ Tarasow TM Phase I study of procaspase activating compound-1 (PAC-1) in the treatment of advanced malignancies. J Clin Oncol. (2016) 34 10.1200/JCO.2016.34.15_suppl.TPS2605 PMC9977881 36470974

[49] Emery IF Gopalan A Wood S Chow KH Battelli C George J. Expression and function of ABCG2 and XIAP in glioblastomas. J Neurooncol. (2017) 133:47–57. 10.1007/s11060-017-2422-z 28432589 PMC5627495

[50] Yang W Cooke M Duckett CS Yang X Dorsey JF. Distinctive effects of the cellular inhibitor of apoptosis protein c-IAP2 through stabilization by XIAP in glioblastoma multiforme cells. Cell Cycle (2014) 13:992–1005. 10.4161/cc.27880 24552816 PMC3984322

[51] Lucas PW Schmit JM Peterson QP West DC Hsu DC Novotny CJ. Pharmacokinetics and derivation of an anticancer dosing regimen for PAC-1, a preferential small molecule activator of procaspase-3, in healthy dogs. Invest New Drugs. (2011) 29:901–11. 10.1007/s10637-010-9445-z 20499133 PMC3182491

[52] Botham RC Fan TM Im I Borst LB Dirikolu L Hergenrother PJ. Dual small-molecule targeting of procaspase-3 dramatically enhances zymogen activation and anticancer activity. J Am Chem Soc. (2014) 136:1312–9. 10.1021/ja4124303 24383395 PMC3954530

[53] Kato Y Nishihara H Mohri H Kanno H Kobayashi H Kimura T. Clinicopathological evaluation of cyclooxygenase-2 expression in meningioma: immunohistochemical analysis of 76 cases of low and high-grade meningioma. Brain Tumor Pathol. (2014) 31:23–30. 10.1007/s10014-012-0127-8 23250387

[54] Rossmeisl JH Jr. Robertson JL Zimmerman KL Higgins MA Geiger DA. Cyclooxygenase-2 (COX-2) expression in canine intracranial meningiomas. Vet Comp Oncol. (2009) 7:173–80. 10.1111/j.1476-5829.2009.00188.x 19691646

[55] York D Higgins RJ LeCouteur RA Wolfe AN Grahn R Olby N. TP53 mutations in canine brain tumors. Vet Pathol. (2012) 49:796–801. 10.1177/0300985811424734 22002975

[56] Dickinson PJ Surace EI Cambell M Higgins RJ Leutenegger CM Bollen AW. Expression of the tumor suppressor genes NF2, 4.1B, and TSLC1 in canine meningiomas. Vet Pathol. (2009) 46:884–92. 10.1354/vp.08-VP-0251-D-FL 19429976

